# Comprehensive transcriptome analysis and flavonoid profiling of Ginkgo leaves reveals flavonoid content alterations in day–night cycles

**DOI:** 10.1371/journal.pone.0193897

**Published:** 2018-03-01

**Authors:** Jun Ni, Lixiang Dong, Zhifang Jiang, Xiuli Yang, Ziying Chen, Yuhuan Wu, Maojun Xu

**Affiliations:** 1 Key Laboratory of Hangzhou City for Quality and Safety of Agricultural Products, College of Life and Environmental Sciences, Hangzhou Normal University, Hangzhou, China; 2 Zhejiang Provincial Key Laboratory for Genetic Improvement and Quality Control of Medicinal Plants, Hangzhou Normal University, Hangzhou, China; Youngstown State University, UNITED STATES

## Abstract

Ginkgo leaves are raw materials for flavonoid extraction. Thus, the timing of their harvest is important to optimize the extraction efficiency, which benefits the pharmaceutical industry. In this research, we compared the transcriptomes of Ginkgo leaves harvested at midday and midnight. The differentially expressed genes with the highest probabilities in each step of flavonoid biosynthesis were down-regulated at midnight. Furthermore, real-time PCR corroborated the transcriptome results, indicating the decrease in flavonoid biosynthesis at midnight. The flavonoid profiles of Ginkgo leaves harvested at midday and midnight were compared, and the total flavonoid content decreased at midnight. A detailed analysis of individual flavonoids showed that most of their contents were decreased by various degrees. Our results indicated that circadian rhythms affected the flavonoid contents in Ginkgo leaves, which provides valuable information for optimizing their harvesting times to benefit the pharmaceutical industry.

## Introduction

*Ginkgo biloba*, a “living fossil”, is a long-lived native Chinese tree species with no living relatives [1]. The extract from Ginkgo leaves, which contains pharmacologically activated flavonoids, is commonly used as an herbal dietary supplement and in the treatment of many diseases [2]. Thus, the extract from Ginkgo leaves has become a popular herbal ingredient in the health product, pharmaceutical, and cosmetic industries.

As a raw material for the pharmaceutical industry, any means to increase the flavonoid contents in Ginkgo leaves are worth pursuing. Thus, a number of studies have been performed to find appropriate ways to achieve this goal. In the Ginkgo cell line, a fungal elicitor induces flavonoid accumulation through a complementary relationship between jasmonic and salicylic acids [3]. Ozone induces flavonoid production through nitrate reductase-mediated nitric oxide signaling [4]. In addition, NaCl treatments can effectively increase the flavonoid content in Ginkgo cells [5]. For the post-harvest Ginkgo leaves, ultraviolet-B and NaCl are effective inducers of flavonoid biosynthesis [6,7].

Compared with the cell line- and post-harvest leaf-associated research, Ginkgo trees attract more attention, and many abiotic factors have positive effects on flavonoid accumulation in Ginkgo trees. Light intensity affects flavonoid biosynthesis in Ginkgo, and full sunlight promotes the expression of flavonoid biosynthesis genes and increases flavonoid biosynthesis [8]. The appropriate air temperature and soil moisture enhance the leaf flavonoid content [9]. Furthermore, alternative partial root-zone irrigation enhances leaf flavonoid accumulation in Ginkgo [10,11]. Interestingly, the variation in the flavonoids’ content with respect to changes in season has also been investigated [12,13].

Most organisms, including plants, change metabolism, physiology and behavior based on their circadian rhythm. Indeed, these organisms do not simply respond to the sunrise and sunset, but adjust their biology according to exogenous time cues from their endogenous circadian clocks [14]. Synchronizing circadian rhythms with daily environmental cycles allows plants to optimize growth and development [15,16,17]. The circadian clock in Arabidopsis is well described, and its mechanism is conserved in other plant species [18]. Recent metabolomic and transcriptomic analyses showed the wide-spread circadian regulation of primary metabolism pathways [19]. However, studies on the circadian-based regulation of secondary metabolism, such as flavonoid metabolism, are still limited.

Here, the circadian rhythm of the flavonoid contents in Ginkgo leaves was investigated. We compared the transcriptomes of Ginkgo leaves harvested at midday and midnight, and found that the flavonoid biosynthesis-related genes were down-regulated at midnight. Consistent with this result, the total flavonoid content was lower at midnight. A high-performance liquid chromatography (HPLC) analysis revealed that different flavonoids varied their contents differently during the day–night cycles. Our results revealed a circadian rhythm for the flavonoid contents in Ginkgo leaves and provides valuable information to optimize flavonoid extraction for the pharmaceutical industry.

## Materials and methods

### Plant materials and RNA extraction

The Ginkgo plants used in this research were grown in the forestry experimental field of Hangzhou Normal University, Hangzhou, China. We harvested the leaves from 20-year-old male trees on sunny days in May. After harvesting Ginkgo leaves at midday (28°C, 1560 μmol·m^−2^·s^−1^) and midnight (19°C, with faint moon light that was undetectable with our photometer), the materials were frozen immediately in liquid nitrogen and stored at −80°C until used. For consistency between gene expression and flavonoid content analyses, the examined leaves were halved before being frozen in liquid nitrogen. Then, one half was used for the gene expression analysis and the other was for the flavonoid content analysis as previously described [7].

RNA was extracted using TRIzol reagent according to the manufacturer’s instructions (Invitrogen, USA). The concentration and integrity of RNA were quantified using an RNA Assay Kit in a Qubit 2.0 Fluorometer (Life Technologies, CA, USA) and RNA Nano 6000 Assay Kit in an Agilent Bioanalyzer 2100 system (Agilent Technologies, CA, USA), respectively.

### Library preparation, deep sequencing and *de novo* assembly

After the extraction of total RNA and digestion of genomic DNA by treating with DNase I, Oligo(dT) was used to isolate mRNA. The mRNA was fragmented by mixing in fragmentation buffer. Then, the corresponding cDNA was synthesized using the mRNA fragments as templates. Short fragments were purified and resolved with EB buffer for end repair and the addition of adenine. The short fragments were then connected with adapters. During the quality control steps, an Agilent 2100 Bioanaylzer and ABI StepOnePlus Real-Time PCR System were used in the quantification and qualification, respectively, of the sample library. Then, the library was sequenced using Illumina HiSeq 4000.

After sequencing, the raw reads that were of low quality, adaptor-polluted or had high contents of unknown bases (more than 5%) were filtered and clean reads remained. The unigenes were assembled *de novo* using these clean reads. We used Trinity (v2.0.6) to perform a *de novo* assembly with the clean reads [20], and then used Tgicl (v2.0.6) to cluster transcripts into unigenes [21]. The raw RNA-seq data can be found at https://www.ncbi.nlm.nih.gov/sra/?term=SRP118730. The assembled unigenes could be found in https://www.ncbi.nlm.nih.gov/nuccore/GGBW00000000.

### Functional annotation of unigenes

The assembled unigenes were used as query against several databases for functional annotation. We used BLAST (v2.2.23) to align unigenes to NT, NR, COG, KEGG, and Swiss-Prot to produce the annotations [22], and Blast2GO (v2.5.0) with the NR annotation to produce the gene ontology (GO) annotation [23], and used InterProScan5 (v5.11–51.0) to produce the InterPro annotation [24].

### Unigene expression and differentially expressed genes (DEG) detection

The quantification of gene expression was based on FPKM. Briefly, the clean reads were mapped to unigenes using Bowtie2 [25], and the gene expression levels were calculated with RSEM [26]. DEGs with fold changes more than two and probabilities > 0.8, unless otherwise specified, were detected with NOIseq. The probability of 0.8 is a default set in NOISeq used to identify DEGs. The probability 0.8 means that the gene is four times more likely to be differentially expressed than nondifferentially expressed [27]. For the functional enrichment analysis, phyper, a function of R, was used. The p value was calculated with hypergeometric test. Then, the false discovery rate for each q value was calculated.

### Quantitative reverse transcription PCR (RT-qPCR)

The reverse transcript reaction was performed with ReverTra Ace qPCR RT Kit (TOYOBO) according to the manufacturer’s instructions. The transcript levels were measured by RT-qPCR using an Mx3000p QPCR System (Agilent) with iQ SYBR Green Supermix (Bio-Rad). The relative expression levels were calculated according to the 2^−ΔΔCt^ method [28,29]. Each experiment was carried out with at least three independent biological replicates. Primer sequences used for RT-qPCR are listed in S1 Table.

### Analysis of flavonoids

The total flavonoid content was measured by the AlCl_3_ colorimetric assay as previously described [30]. The extraction and HPLC analysis of flavonoids were carried out as previously described with minor modifications [7]. Briefly, equal amounts of Ginkgo leaves were freeze-dried. In each experiment, 50 mg of frozen powder was extracted in 1.5 ml extraction solvent (methanol:acetate:H_2_O, 9:1:10) at 37°C for 30 min. After centrifugation at 14,000 *g*, the supernatant was filtered through a 0.25-μm membrane. Then, 10 μl of supernatant was applied to a Waters HPLC (e2695 series). HPLC was carried out on an XBridge C18 (Φ 4.6 mm × 250 mm) at a flow rate of 0.8 ml·min^-1^. An elution gradient with solvent A (CH_3_CN–H_2_O–TFA, 10:90:0.1) and solvent B (CH_3_CN–H_2_O–TFA, 90:10:0.1) were used in the following elution profile: 0 min, 100% solvent A; 30 min, 70% solvent A; 32 min, 0% solvent A; 33 min, 0% solvent A; and 35 min, 100% solvent A with linear gradients in between the time points. The column temperature was set to 40°C. The changes in individual flavonoid contents were calculated by comparing the areas of individual peaks. PDA was used for the detection of UV-visible absorption in the range of 190–510 nm. Flavonoids were detected at 360 nm.

## Results

### Sequencing and sequence assembly of the transcriptome from Ginkgo leaves

To investigate the diurnal variation of the transcriptome from Ginkgo leaves, total RNAs were extracted and isolated from Ginkgo leaves at midday and midnight. The subsequent cDNA libraries, which were reverse-transcribed from RNAs, were subjected to high-throughput parallel sequencing using an Illumina HiSeq platform. A total of 268.63 million (M) clean reads with 40.3 Gb clean bases were obtained after removing adaptor sequences and low-quality reads. In addition, the ratios of clean reads in each sample were more than 90%, indicating that the quality of the sequencing data was acceptable (Table 1). Using the Trinity assembly program [20], these clean reads were assembled into 439,885 transcripts, with a median length of more than 850 bp and an N50 of ~1,700 bp (S2 Table). These transcripts were analyzed with Tgicl [21] to generate 80,765 unigenes with a mean length of 1,208 bp and an N50 of 2,251 bp (Table 2). Unigenes with lengths ranging from 300–1000 bp, 1000–2000 bp, and 2000–3000 bp accounted for 59.07% (47,706), 19.74% (15,941), and 11.92% (9,627) of the total, respectively. In addition, 7,491 (9.28%) unigenes were longer than 3,000 bp (S1 Fig).

**Table 1:** Summary of sequencing reads after filtering. D-1, D-2 and D-3 are three biological repeats at midday. N-1, N-2 and N-3 are three biological repeat at midnight. Q20: the percentage of bases with a Phred value more than 20; Q30: the percentage of bases with a Phred value more than 30.

Sample	Total Raw Reads(M)	Total Clean Reads(M)	Total Clean Bases(Gb)	Clean Reads Q20(%)	Clean Reads Q30(%)	Clean Reads Ratio(%)
**D-1**	48.46	44.97	6.75	95.84	91.37	92.79
**D-2**	48.48	44.39	6.66	95.38	90.63	91.57
**D-3**	48.5	45.1	6.77	95.97	91.6	93
**N-1**	47.35	44.19	6.63	95.61	90.94	93.31
**N-2**	48.98	45.29	6.79	95.73	91.19	92.47
**N-3**	47.35	44.69	6.7	95.72	91.1	94.38

**Table 2:** Quality metrics of unigenes. N50: a weighted median statistic in which 50% of the total length is contained in unigenes greater than or equal to this value. GC (%): the percentage of G and C bases in all unigenes.

Sample	Total Number	Mean Length	N50	N70	N90	GC(%)
**D-1**	46431	1078	1883	1237	417	42.14
**D-2**	49675	1095	1914	1257	426	41.96
**D-3**	52513	1075	1943	1240	397	41.84
**N-1**	49724	1070	1895	1224	407	42.18
**N-2**	51530	1105	1976	1275	421	41.86
**N-3**	48419	1089	1904	1254	422	42.25
**All-Unigene**	80765	1208	2251	1508	467	41.81

### Functional annotations and classifications of predicted proteins

Functional annotations of predicted proteins encoded by these unigenes were performed using seven protein databases (NR, NT, GO, COG, KEGG, Swiss-Prot, and InterPro). Of the 80,765 unigenes, 47,436 unigenes were annotated in NR, which accounted for 58.73% of the unigenes. For other databases, 36,990 unigenes were annotated in NT (45.80%), 34,264 unigenes in Swiss-Prot (42.42%), 36.957 in KEGG (45.76%), 21,482 in COG (26.61%), 38,941 in InterPro (48.22%), and 8,501 in GO (10.53%). Overall, 50,981 unigenes were annotated in at least one of the seven databases, which accounted for 63.12% of the total unigenes (Table 3 and S3 Table). We also created a Venn diagram, and found that 16,393 unigenes (20.30%) were annotated by NR, COG, KEGG, Swiss-Prot, and InterPro simultaneously (S2 Fig).

**Table 3:** Summary of functional annotations against major public databases. Overall: the number of unigenes which can be annotated in at least one functional database.

Databases	Number of unigenes	Percentage (%)
**NR**	47,436	58.73
**NT**	36,990	45.80
**Swissprot**	34,264	42.42
**KEGG**	36,957	45.76
**COG**	21,493	26.61
**Interpro**	38,941	48.22
**GO**	8,501	10.53
**Overall**	50,981	63.12

### Functional classification of the transcriptome

To identify biological pathways in Ginkgo leaves, unigenes were queried against the KEGG database. The most represented pathways were metabolic pathways (21,688; 58.47%), including amino acid metabolism (1,764; 4.76%), biosynthesis of other secondary metabolites (1,416; 3.82%), carbohydrate metabolism (3,065; 8.26%), energy metabolism (1,173; 3.16%), global and overview maps (8,025; 21.63%), glycan biosynthesis and metabolism (711; 1.92%), lipid metabolism (2,091; 56.37%), metabolism of cofactors and vitamins (931; 2.51%), metabolism of other amino acids (872; 2.35%), metabolism of terpenoids and polyketides (831; 2.24%), and nucleotide metabolism (809; 2.18%).

**Fig 1:**
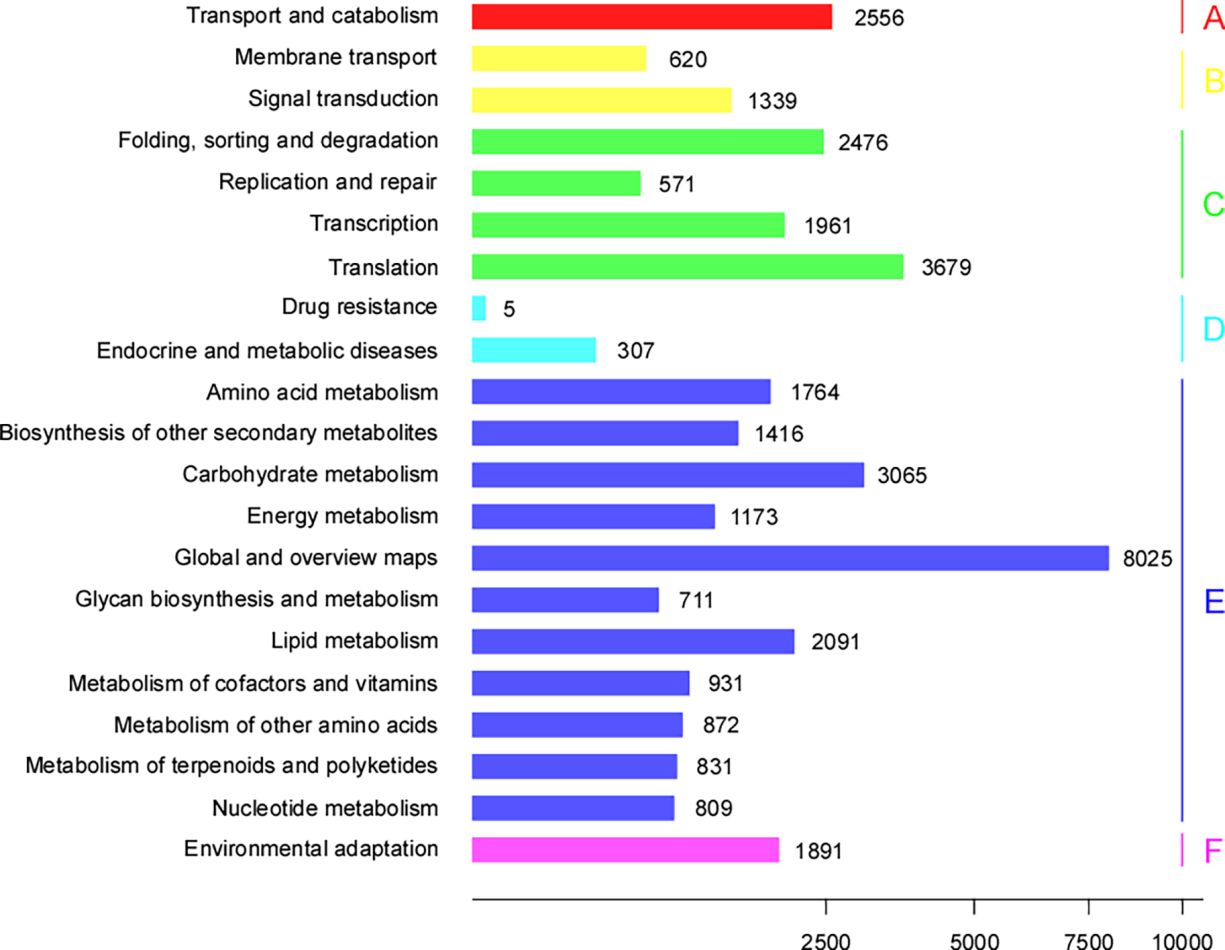
Functional distribution of the KEGG annotation. X-axis represents the numbers of unigenes. Y-axis represents the KEGG functional category. A, cellular processes; B, environmental information processing; C, genetic information processing; D, human diseases; E, metabolism; and F, organismal systems.

In addition, 8,687 unigenes (23.42%) were matched to genetic information processing, including folding, sorting and degradation (2,476; 6.68%), replication and repair (571; 1.54%), transcription (1961; 5.29%), and translation (3,679; 9.92%). Finally, many unigenes were also involved in transport and catabolism (2,556; 6.89%), environmental adaptation (1,891; 5.10%), membrane transport and signal transduction (1,959; 5.28%), and drug resistance, endocrine and metabolic diseases (312; 0.84%) (Fig 1 and S4 Table).

We also performed a GO classification analysis and found that 16,827 unigenes were involved in biological process, 13,425 unigenes were involved in cellular components, and 10,067 unigenes were involved in molecular function (S3 Fig and S5 Table).

The annotation of the unigenes expressed in Ginkgo leaves provided valuable information for investigating specific processes and pathways. Furthermore, the data allowed for the further analysis of the diurnal variation of the transcriptome in Ginkgo leaves.

### Analysis of DEGs

To investigate the repeatability of our experiment, we compared the expressed unigenes of three biological repeats from midday and midnight. We found that 38,908 unigenes were detected in all three biological repeats at midday, which accounted for 66.14% of the total unigenes detected at midday. In addition, 39,189 unigenes were detected in the three biological repeats at midnight, which accounted for 65.59% of the total unigenes detected at midnight (S4 Fig). The expression levels of these midday- and midnight-expressed unigenes with good repeatability were compared with each other, and the DEGs were identified.

A total of 353 DEGs were identified as having diurnal variations (S6 and S7 Tables). Compared with midday-expressed unigenes, the expression levels of 211 DEGs were up-regulated and those of 142 DEGs were down-regulated at midnight (S5 Fig). A GO classification analysis showed that 108 DEGs were involved in biological process, 155 DEGs were involved in cellular component, and 60 DEGs in molecular function (S6 Fig). A detailed analysis showed that DEGs in translation (GO: 0006412) and electron transport chain (GO: 0022900) were enriched in biological process (S7 Fig), intracellular ribonucleoprotein complex (GO: 0030529) was enriched in cellular component (S8 Fig), and heme-copper terminal oxidase activity (GO: 0015002) was enriched in molecular function (S9 Fig).

All of the DEGs were then mapped to the KEGG database to identify genes involved in signal transduction or metabolic pathways. A great majority of the DEGs were involved in metabolism, including amino acid metabolism (9), biosynthesis of other secondary metabolites (4), carbohydrate metabolism (7), energy metabolism (20), global and overview maps (36), glycan biosynthesis and metabolism (2), lipid metabolism (4), metabolism of other amino acids (2), metabolism of terpenoids and polyketides (2), and nucleotide metabolism (2). Many DEGs were also involved in genetic information processing, including folding, sorting and degradation (12), replication and repair (2), transcription (7), and translation (54). In addition, some DEGs were involved in other processes or metabolic pathways, including transport and catabolism (8), membrane transport (1), signal transduction (7), and environmental adaptation (11) (S10 Fig).

**Fig 2:**
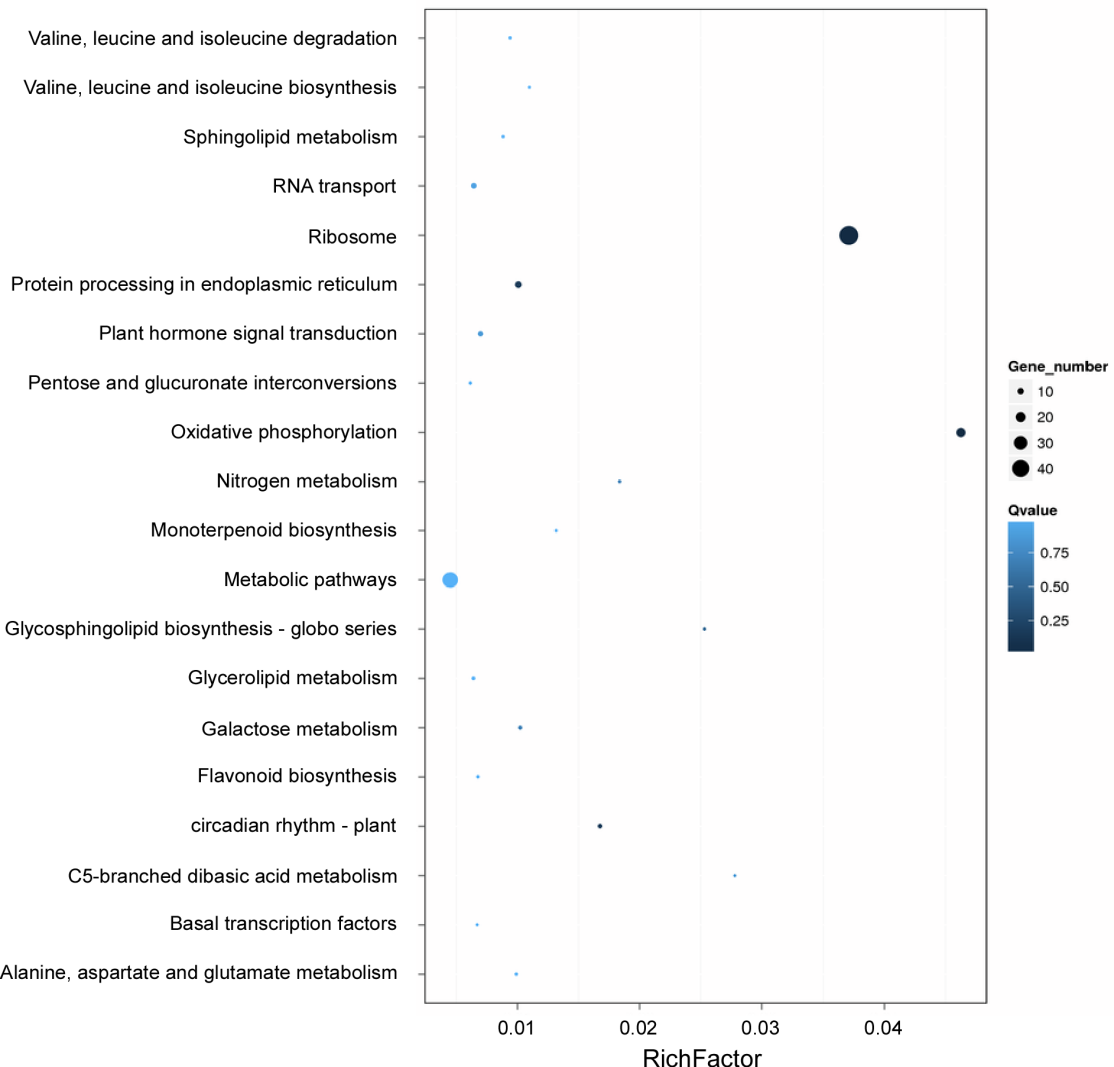
Pathways functionally enriched with DEGs. X-axis represents the enrichment factor. Y-axis represents the pathway name. Coloring correlates with the q-value. The lower the q-value, the more significant the enrichment. Point size correlates with the number of DEGs.

A KEGG enrichment analysis of the DEGs showed that genes involved in the oxidative phosphorylation were the most enriched. Many genes involved with the ribosome were also greatly enriched. The q values of these two pathways are low, indicating the significant enrichment of DEGs in oxidative phosphorylation and ribosome. For some pathways, such as nitrogen metabolism, glycosphingolipid biosynthesis, circadian rhythm, and C5-branched dibasic acid metabolism, although the gene numbers were limited, the enrichment factors were high (Fig 2 and S8 Table).

### The expression levels of flavonoid biosynthesis-related genes varied during the day–night cycle

Based on the strict requirements described previously, only one flavonoid biosynthesis-related gene was identified as being down-regulated at midnight. The gene was *CL2379*.*Contig4_All*, which was similar to flavanone 3-hydroxylase (S7 Table). Because many flavonoid biosynthesis-related genes were identified in this research (S11 Fig), we lowered the criteria and searched for gene changes that had a probability greater than 0.5, rather than 0.8, to fully understand the expression levels of flavonoid biosynthesis-related genes during the day–night cycle.

We identified 6 *CHS*-like genes, and 5 of the 6 genes were down-regulated at midnight. Although one gene was up-regulated by more than 100-fold at midnight, the relative repression level was much lower than those of other genes. We identified one *CHI*-like gene with a probability of more than 0.5, and its expression level was down-regulated at midnight. We also identified 5 flavanone 3-hydroxylase-like genes, of which 4 were down-regulated at midnight. For other genes, the situation was rather complex. For the 13 *FLS*-like genes, 5 genes were down-regulated, while 8 genes were up-regulated. For the 19 *F3’H*-like genes, 8 genes were down-regulated compared with 11 up-regulated genes. For the 2 *DFR*-like genes, one gene was up-regulated and the other gene was down-regulated. (S9 Table).

**Fig 3:**
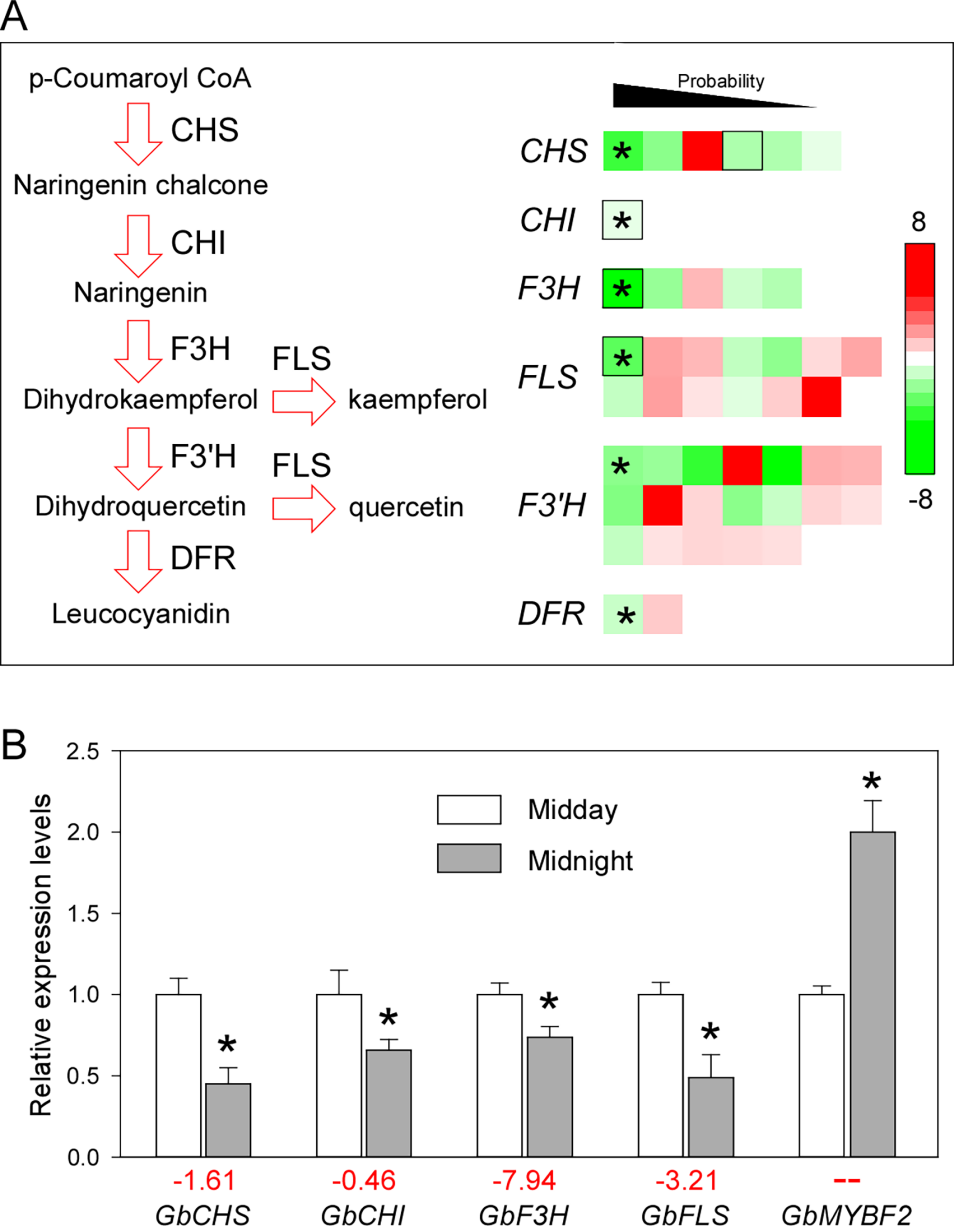
The expression levels of flavonoid biosynthesis-related genes. **(A)** Diagram of flavonoid biosynthesis and the expression changes of corresponding genes according to the sequencing analysis. The different colors correspond to the log2-fold change of gene expression levels. Asterisks indicate the gene expressions with the highest probabilities. Unigenes validated by RT-qPCR are marked with black boxes. **(B)** Relative expression levels of flavonoid biosynthesis-related genes. Asterisks indicate significant differences (*P* < 0.01; Student’s *t*-test). Red numbers under the bar graphs are log2-fold change values of each gene acquired in the transcriptome analysis. Three independent replicates were performed for midday and midnight (mean ± SD).

To further investigate the reliability of these gene expression levels, we ranked the genes in descending order by their probability values and found that the genes with the highest probabilities in each group showed decreased expression levels at midnight (Fig 3A). This indicated that although many flavonoid biosynthesis-related genes showed up-regulated expression levels at midnight according to the transcriptome analysis, the probability values were low. Thus, it is possible that the flavonoid biosynthesis-related genes are down-regulated at midnight.

### RT-qPCR validation of flavonoid biosynthesis-related gene expression levels

We also used real-time PCR to examine the expression levels of flavonoid biosynthesis-related genes at midday and midnight. To exclude the inference of the transcriptome results, we examined genes that were characterized previously, independent of the transcriptome results [31,32,33,34]. As expected, the expression levels of the examined structural genes were down-regulated, which was similar to the transcriptome results having the highest probabilities. In addition, the expression of *GbMYBF2*, a negative regulator of flavonoid biosynthesis [35], was up-regulated at midnight (Fig 3B). In addition, we also verified the expression levels of flavonoid biosynthesis-related genes that were not characterized previously, and the RT-qPCR results were similar to the transcriptome results (S12 Fig). These results confirmed that flavonoid biosynthesis-related genes were down-regulated at midnight.

### The flavonoids contents were lower at midnight than at midday

**Fig 4:**
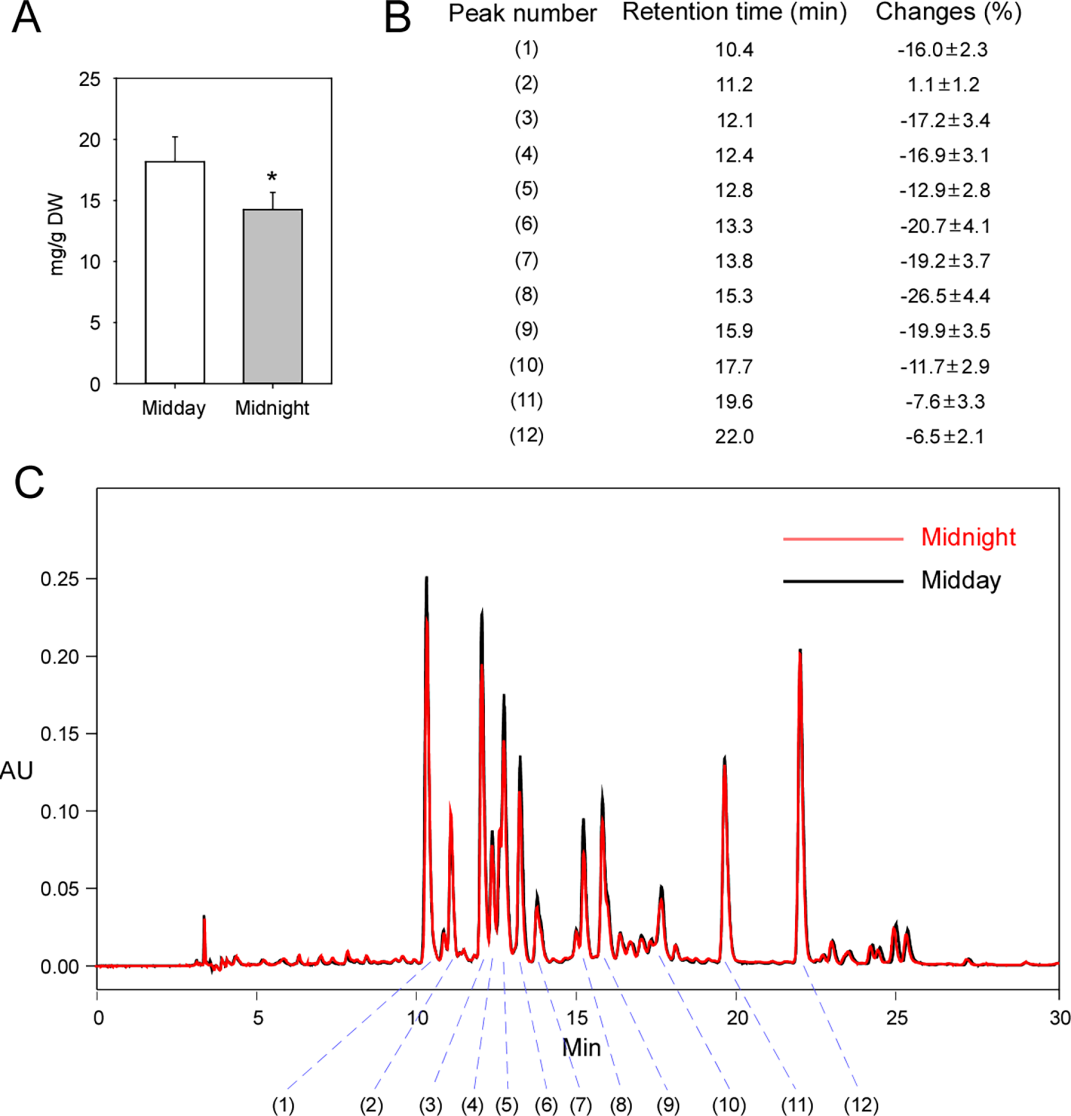
A comparison of the flavonoid contents between midday and midnight. **(A)** A comparison of the total flavonoid contents of Ginkgo leaves harvested at midday and midnight (*P* < 0.01; Student’s *t*-test). **(B)** Changes in different flavonoid contents separated using the HPLC method. **(C)** Typical HPLC fingerprints (absorption at the 360-nm wavelength) of the total extract of Ginkgo leaves harvested at midday (black) and midnight (red). The main peaks are indicated by numbers. Asterisks indicate significant differences. Three independent replicates were performed for midday and midnight (mean ± SD).

To investigate the changes in flavonoid contents during the day–night cycles, we measured the total flavonoid contents at midday and midnight. The total flavonoid content was significantly lower at midnight compared with at midday (Fig 4A). To further investigate the changes in individual flavonoids during the day–night cycles, we used HPLC to separate and measure the different flavonoids found in Ginkgo leaves. The HPLC fingerprint of the total extract showed several peaks, and we chose 12 major peaks (indicated by No. 1–12) for further analysis (Fig 4B and 4C). UV absorption spectral analyses of these peaks showed that, except for nonspecific absorption near 200 nm that was probably caused by the solvent or other impurities, all of the peaks exhibited two major absorption bands in the UV region. Furthermore, 9 (No. 1, and 3–10) of the 12 peaks had first absorption bands near 260 nm and second absorption bands near 350 nm, which is in accordance with the characteristic absorption spectrum of a flavonoid [36], indicating that most of the peaks analyzed were flavonoids (S13 Fig). We compared the areas under the 12 peaks and found that all of the areas, except for that of No. 2, decreased, indicating an overall decrease in most flavonoids at midnight. Then, we compared the change rates among the 12 peaks and found that 9 peaks (No. 1, and 3–10) had rates that decreased by more than 10% at midnight. Coincidently, each of these peaks had the characteristic absorption spectrum of a flavonoid. However, two peaks (No. 11 and 12) decreased by less than 10% at midnight, and peak No. 2 increased, although not significantly (Fig 4B).

## Discussion

Ginkgo is not only a traditional Chinese medicine, but also acts as a raw material for modern pharmaceutical industries [37]. Because plants utilize circadian clocks to synchronize their physiological and developmental events with daily environmental changes [18], it is important to investigate whether the timing of harvesting affects the quality of the final pharmaceutical products. Thus, we first compared the transcriptomes of Ginkgo leaves harvested at midday and midnight.

Recently, with the development of next-generation sequencing technology, a number of transcriptome analyses in Ginkgo have been published [38,39,40]. However, these focused on the transcriptomes of sterile seedlings [40], kernels [38], and different sexual types [39] of Ginkgo. The transcriptome of the mature Ginkgo leaf, which is the most important organ for pharmaceutical industries, was not investigated. In addition, due to the rapid development of next-generation sequencing technology, our sequencing data was of higher quality than those previously published. For example, previously, the mean length of the assembled unigene was ~800 bp, with an N50 value of less than 2,000 bp. In our experiment, the mean unigene length was 1,208 bp, with an N50 value of 2,251 bp. Thus, our sequencing data extended the scope and depth of transcriptome research in Ginkgo and provides a more reliable bioinformatics resource for studies on gene cloning and other molecular research in Ginkgo.

In our research, 80,765 unigenes were identified, and 50,981 unigenes were annotated in at least one database, which accounted for 63.12% of the total unigenes. The number of unigenes identified was more than twice that of some previous studies [38,40], but less than a previous study [39]. Because these studies utilized different plant tissues, the quality of the sequence data cannot be judged simply by the number of identified unigenes. In our research, we used leaves for the transcriptome analysis, while Du and colleagues, for example, used female buds, male buds, ovulate strobilus, and staminate strobilus for the transcriptome analysis [39]. The proportion of annotated genes in our research is the highest among all of the transcriptome analyses performed in Ginkgo. This may be due to the use of different plant parts for sequencing in different studies.

We identified 211 up-regulated and 142 down-regulated DEGs in the transcriptome analysis, which accounted for only 0.44% (353/80,765) of the identified genes. The percentage of circadian-regulated genes was much lower than expected. The integration of multiple circadian microarray experiments showed that approximately one-third of expressed Arabidopsis genes are circadian regulated [41]. We believe that the number of identified DEGs was limited for several reasons. First, the DEG identification parameters were restricted. Only genes with fold changes of more than 2 and probabilities greater than 0.8 were identified as DEGs [27]. We harvested the sequencing samples (Ginkgo leaves) from the perennial Ginkgo trees in the field. Thus, the status of individual samples in biological repeats may be quite different. As expected, only ~65% of the genes were detected in all three biological replicates. As a result, although we identified many DEGs, a large proportion of the differences had probabilities less than the threshold value (0.8). Second, we only chose two time points for the analysis (midday and midnight). Many genes that had different expression levels at other time points would not have been identified. For instance, *CIRCADIAN CLOCK ASSOCIATED 1* (*CCA1*) is regulated by circadian rhythms and is considered a key regulator of the circadian clock in plants [42]. Because of the conserved mechanism of circadian clocks in the plant kingdom, CCA1 should also play similar roles in Ginkgo leaves [18]. However, we failed to find homologs of the *CCA1* gene among the 353 DEGs. In addition, because *CCA1* had its maximum expression level near dawn [43], the difference between midday and midnight may not be significant. Third, because Ginkgo is one of the oldest living plant species and is often referred to as a “living fossil” [44], it is possible that the number of circadian-regulated genes is small and that the circadian-regulated system is simpler than Arabidopsis. In the green unicellular alga *Ostreococcus tauri*, two master clock genes, *TIMING OF CAB EXPRESSION1* (*TOC1*) and *CCA1*, appear to be conserved, while many other genes are lacking [45].

The growth conditions in the field differ in many aspects during the day–night cycles. The most obvious differences are in the light intensity and temperature between midday and midnight. Under normal conditions, both the light intensity and temperature are higher at midday than midnight. Light intensity is an important environmental factor in the induction of flavonoid biosynthesis [8,46]. However, low temperature is also an inducer of flavonoid accumulation [47]. Thus, it is not easy to determine which factor has the main role. In our research, the total flavonoid content decreased at midnight. Thus, although we found various expression changes in flavonoid biosynthesis-related genes, all of the genes with the highest probabilities in each step were down-regulated at midnight. In addition, the real-time PCR validation obtained similar results. Furthermore, *GbMYBF2*, which encodes a negative regulator of flavonoid biosynthesis in Ginkgo leaves [35] was up-regulated. We searched the unigene corresponding to *GbMYBF2* in the RNA-seq data and found that the probability was too low. Thus, we do not further discuss *GbMYBF2* from our RNA-seq data.

Compared with the single-copy genes in Arabidopsis, Ginkgo may employ multigene families to control each step of the flavonoid biosynthesis pathway, resulting in a complex network [48]. A Southern blot analysis of *GbCHS* resulted in several bands, indicating the existence of a multigene *GbCHS* family in Ginkgo [49]. The whole genome sequence of Ginkgo indicated an expansion of the genes involved in flavonoid biosynthesis [50]. Although the number of flavonoid biosynthesis-related genes is still unknown, our transcriptome results showed numerous assembled putative genes. This provides new evidence that further supports the existence of multigene families that control flavonoid biosynthesis in Ginkgo. In our experiment, we found several genes involved in each step. Although the genes with the highest probabilities showed decreased expression levels at midnight, some genes had increased expression levels at midnight. For example, there was an increase in the flavonoid content corresponding to peak No. 2 at midnight, although it was not significant. Thus, we proposed that on the whole, the gene expression levels and total flavonoid contents are lower at midnight. However, for individual genes and flavonoids, the situations are complex. Alternatively, the flavonoid content might not be directly reflected by gene expression abundance. Flavonoid compounds can be depredated, stored, transported or utilized, which might result in the differential flavonoid composition during the diurnal cycle.

Because different flavonoids may show various pharmacological activities [51], it is more important to investigate the changes in individual flavonoids during the day–night cycles. Although more than 70 flavonoids were estimated to exist in Ginkgo [36], it is not easy to analyze all of these flavonoids. As a result, we chose 12 main peaks in the HPLC fingerprint for further analyses. Of these peaks, three (No. 2, 11, and 12) showed atypical UV absorption spectra. However, we could not simply conclude that they were not flavonoids. In previous research, the structures of several peaks in an HPLC fingerprint were identified, and our HPLC fingerprint was very similar to the previous one [51]. Thus, peaks No. 11 and 12 are very likely to be quercetin 3-*O*-α-(6”‘-p-coumaroyl glucopyranosyl-β-1,2-rhamnopyranoside) and kaempferol 3-*O*-α-(6”‘-p-coumaroyl glucopyranosyl-β-1,2-rhamnopyranoside), respectively, which are flavonoids with complex structures. Diurnal changes in flavonoids were observed very early in plants [52]. Plants accumulate flavonoids rapidly to prevent from the enhanced solar UV-B radiation during the morning [53]. Further research revealed that this phenomenon is widespread among higher plants [54]. In this research, diurnal changes in the total flavonoid content were also observed in Ginkgo leaves. Furthermore, we examined individual flavonoid content changes in day–night cycles. Although most of the flavonoids examined showed relatively lower contents at midnight, the degrees were different. The No. 8 peak showed the maximum changes in the day–night cycle, which indicated that this kind of flavonoid was the most sensitive to the circadian rhythms. In the contrast, the No. 2 peak did not show significant changes. Our results are valuable to the pharmaceutical industry, because if flavonoid levels are sensitive to circadian rhythms, then the timing of the harvest should be restricted. Thus, the sensitive flavonoids’ contents would be lower if the leaves were harvested at the wrong time points. In contrast, for flavonoids that were insensitive to circadian rhythm, the timing of the harvest could be flexible. Thus, the process of harvesting should be different depending on the properties of the flavonoids desired by the pharmaceutical industry. In this research, to observe the most significant changes, only two time points were investigated (midday and midnight). It is a common practice that herbs are harvested in the morning. Thus, in further research that may be beneficial to the pharmaceutical industry, it will be necessary to measure shorter time intervals.

## Supporting information

S1 Fig Length distribution of assembled unigenes. X-axis represents the lengths of unigenes, Y-axis represents the numbers of unigenes.
(TIF)

S2 Fig Venn diagram of unigenes identified in the NR, COG, KEGG, Swiss-Prot and InterPro databases. (TIF)

S3 Fig Functional distribution of the GO annotation. The X-axis represents the numbers of unigenes. The Y-axis represents the GO category.
(PDF)

S4 Fig **Venn diagram of expressed genes at midday (A) and midnight (B).** Data represent three biological replicates.
(TIF)

S5 Fig MA plot of DEGs. X-axis represents value A (log2 transformed mean expression level). Y-axis represents value M (log2 transformed fold change). Red points indicate up-regulated DEGs. Blue points indicate down-regulated DEGs. Black points indicate non-DEGs.
(TIF)

S6 Fig GO classifications of DEGs. (TIF)

S7 Fig GO functional enrichment of DEGs in biological process. Coloring correlates with q-value. The lower the q-value, the more significant the enrichment.
(PDF)

S8 Fig GO functional enrichment of DEGs in cellular component. (PDF)

S9 Fig GO functional enrichment of DEGs in molecular function. (PDF)

S10 Fig KEGG pathway classifications of DEGs. (PDF)

S11 Fig Flavonoid biosynthesis pathways in Ginkgo. Steps identified in our transcriptome analysis are marked by red boxes.
(TIF)

S12 Fig RT-qPCR validation of genes identified in the transcriptome analysis. Asterisks indicate significant differences (*P* < 0.01; Student’s *t*-test). Red numbers under the bar graphs are log2-fold change values of each gene acquired in the transcriptome analysis. Three independent replicates were performed for midday and midnight (mean ± SD).
(TIF)

S13 Fig UV absorption spectral analysis of 12 peaks selected in the fingerprint. The wavelengths of two absorption peaks are marked.
(TIF)

S1 Table Primers used in RT-qPCR. (PDF)

S2 Table Quality metrics of transcripts. (PDF)

S3 Table The summarized annotation results. NA: not annotated in this database.
(XLSX)

S4 Table KEGG annotations of unigenes. (XLSX)

S5 Table GO annotations of unigenes. (XLSX)

S6 Table The expression analysis of the unigenes. (XLSX)

S7 Table Summary of DEGs. (XLSX)

S8 Table KEGG enrichment of DEGs. (XLSX)

S9 Table Summary of DEGs involved in flavonoid biosynthesis (probability greater than 0.5) and their sequences. (XLSX)
