# Supplementary figures and images for: Comprehensive transcriptome analysis and flavonoid profiling of Ginkgo leaves reveals flavonoid content alterations in day–night cycles
Source: PLoS One. 2018 Mar 1;13(3):e0193897. doi: 10.1371/journal.pone.0193897 (PMC5833276; doi:10.1371/journal.pone.0193897)

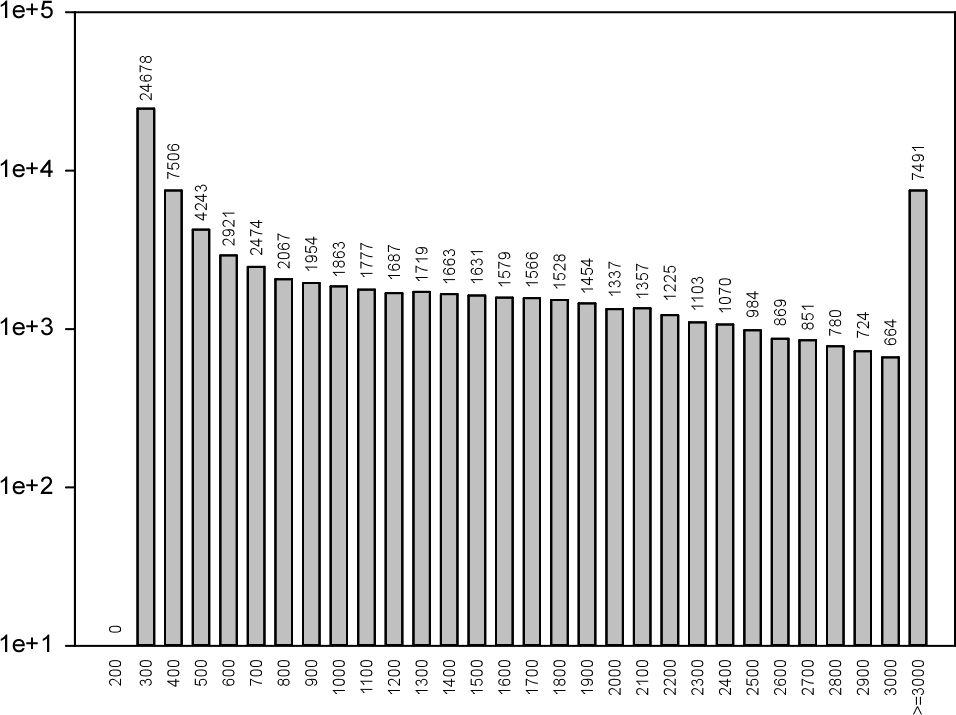

Supplement: S1 Fig — X-axis represents the lengths of unigenes, Y-axis represents the numbers of unigenes. (TIF) [file pone.0193897.s001.tif]

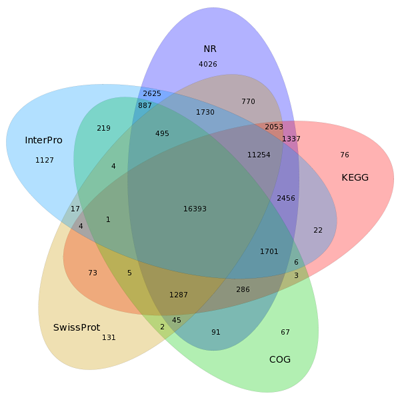

Supplement: S2 Fig — (TIF) [file pone.0193897.s002.tif]

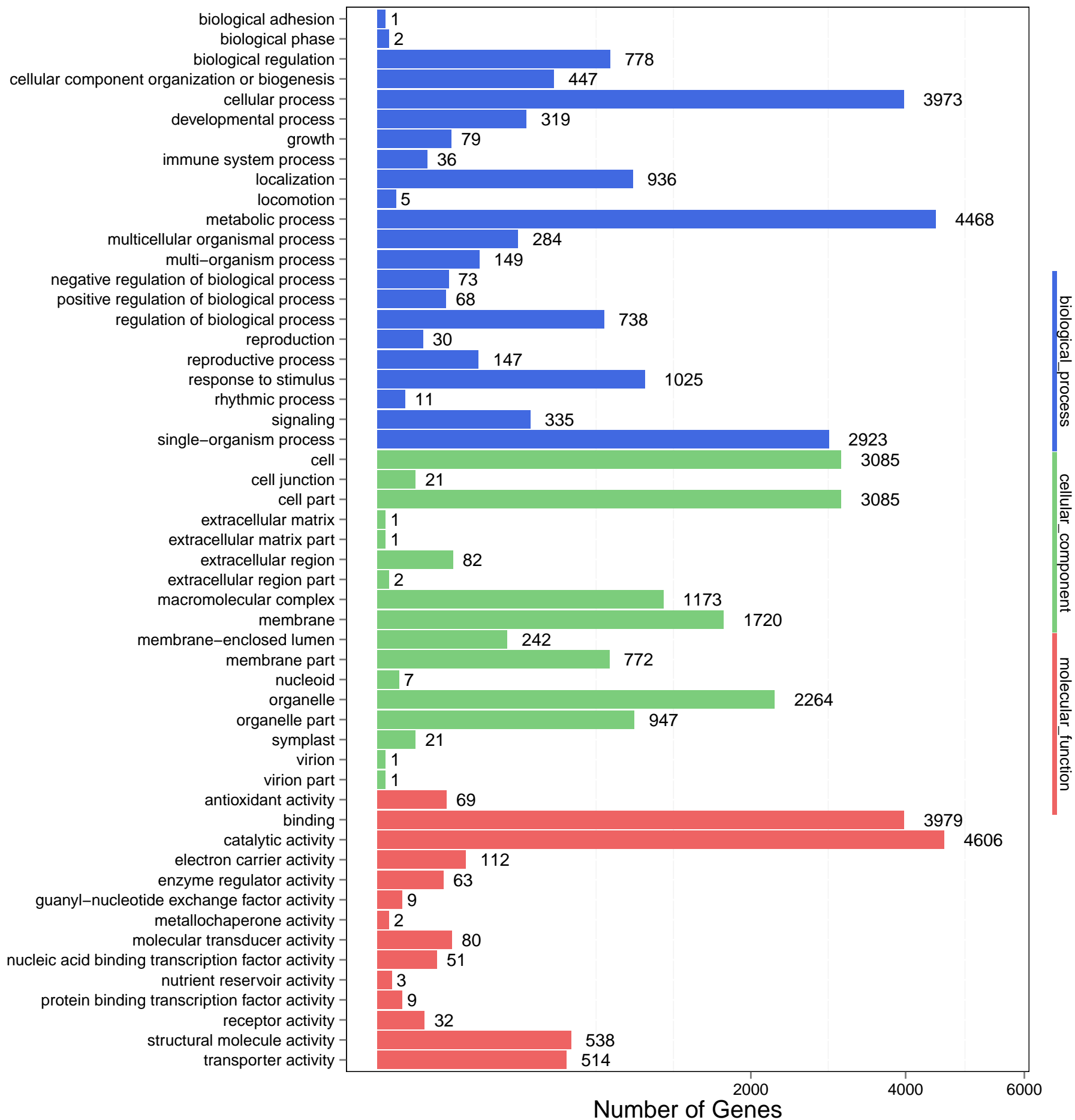

Supplement: S3 Fig — The X-axis represents the numbers of unigenes. The Y-axis represents the GO category. (PDF) [file pone.0193897.s003.pdf]

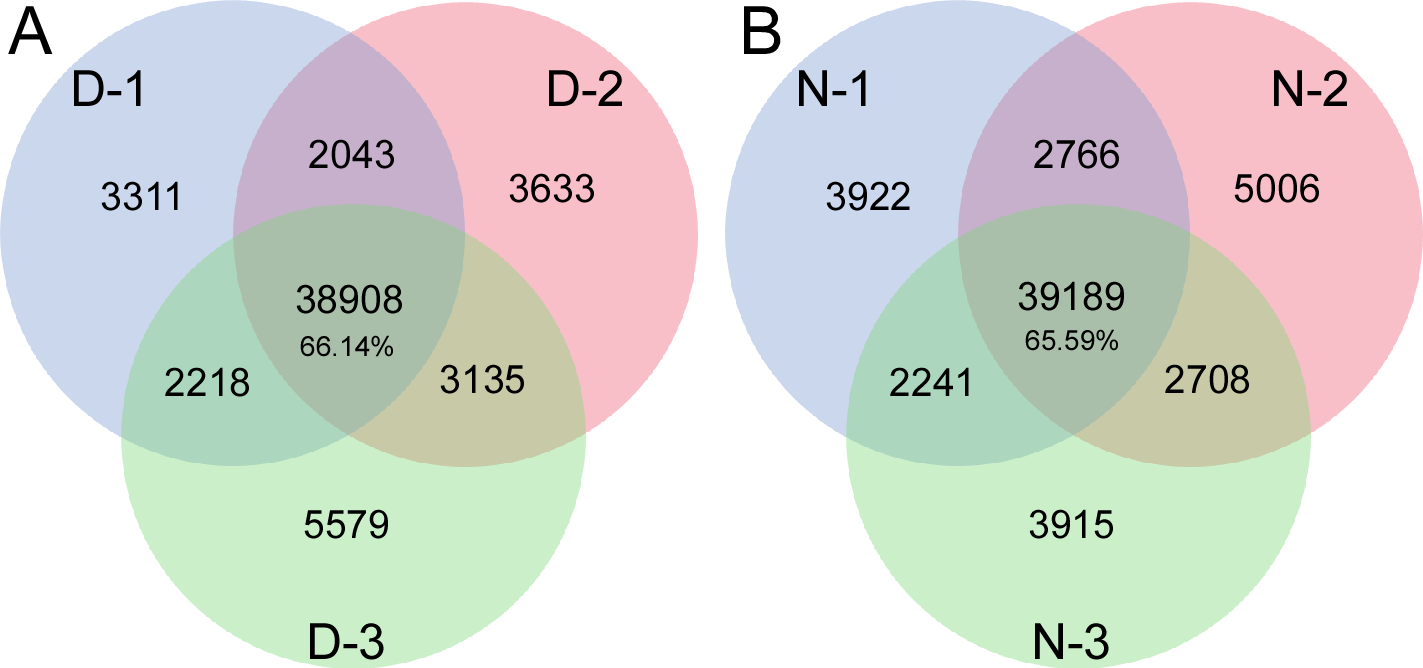

Supplement: S4 Fig — Venn diagram of expressed genes at midday (A) and midnight (B). Data represent three biological replicates. (TIF) [file pone.0193897.s004.tif]

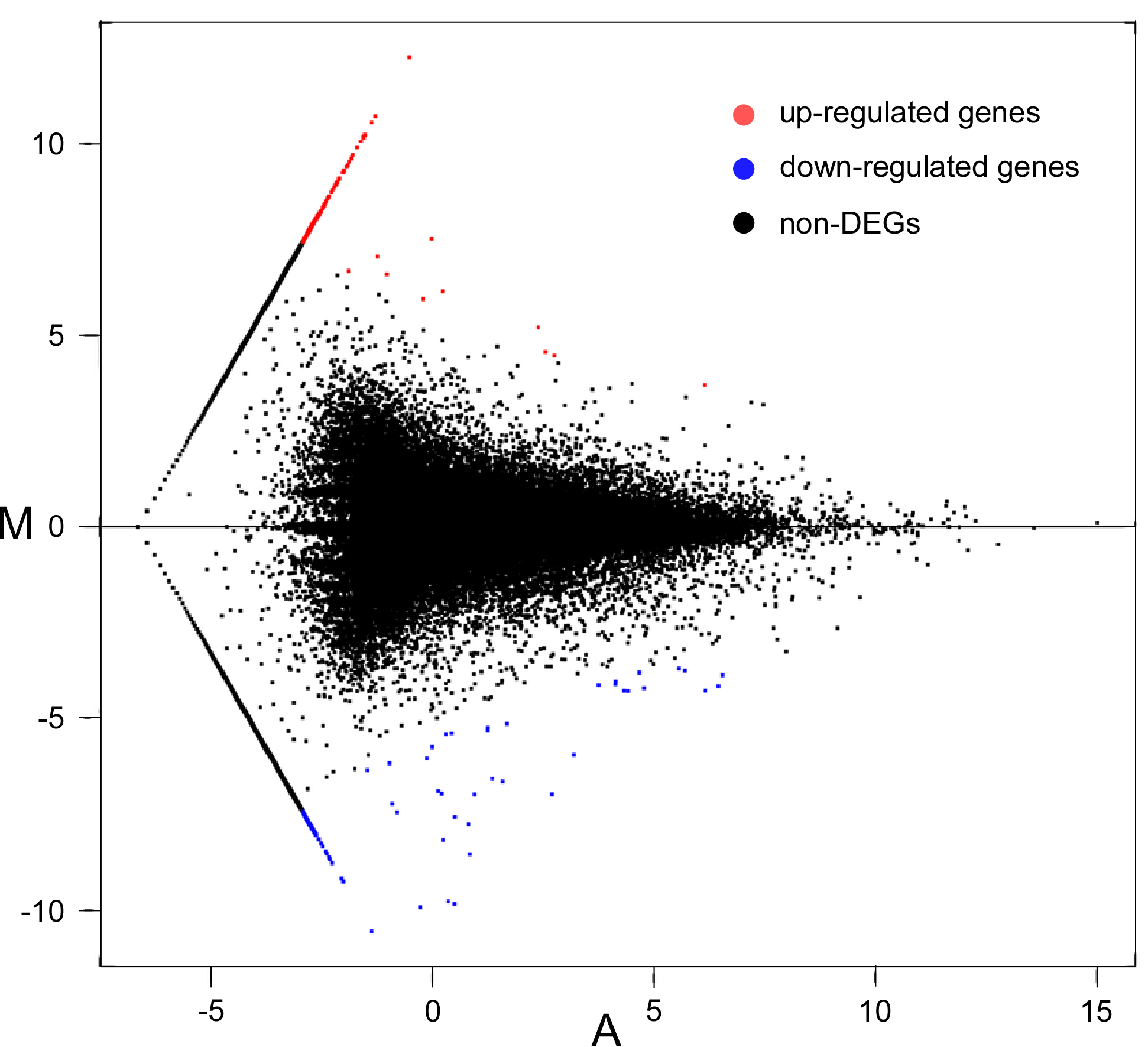

Supplement: S5 Fig — Y-axis represents value M (log2 transformed fold change). Red points indicate up-regulated DEGs. Blue points indicate down-regulated DEGs. Black points indicate non-DEGs. (TIF) [file pone.0193897.s005.tif]

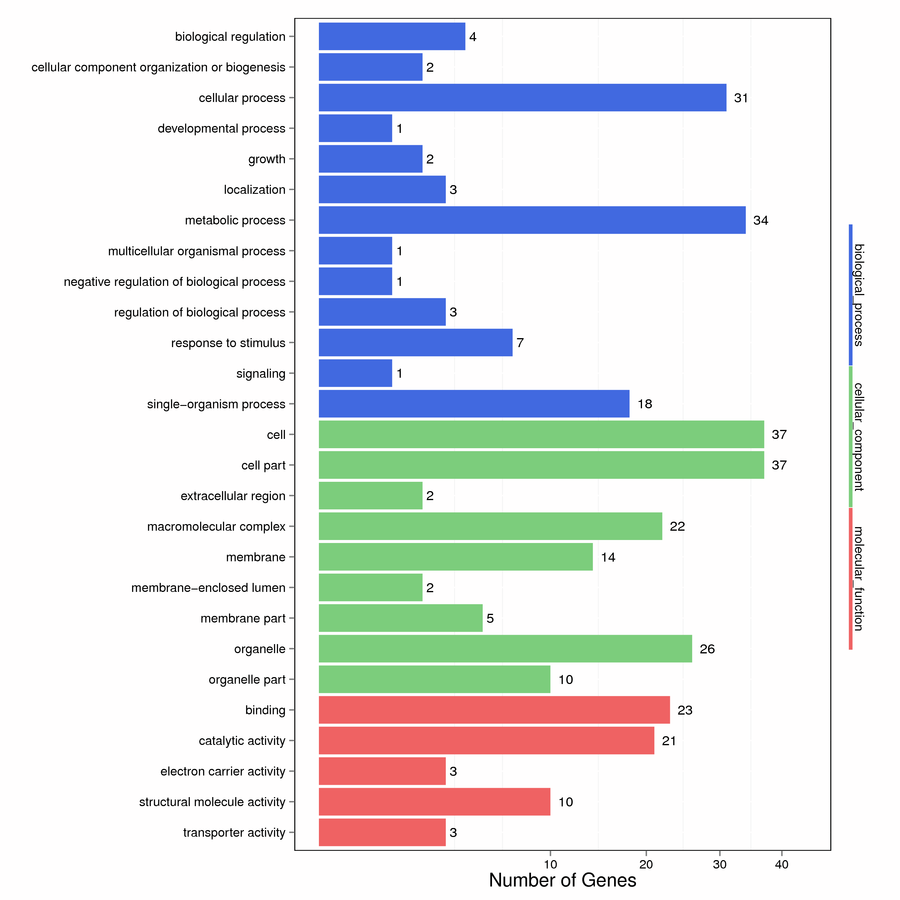

Supplement: S6 Fig — (TIF) [file pone.0193897.s006.tif]

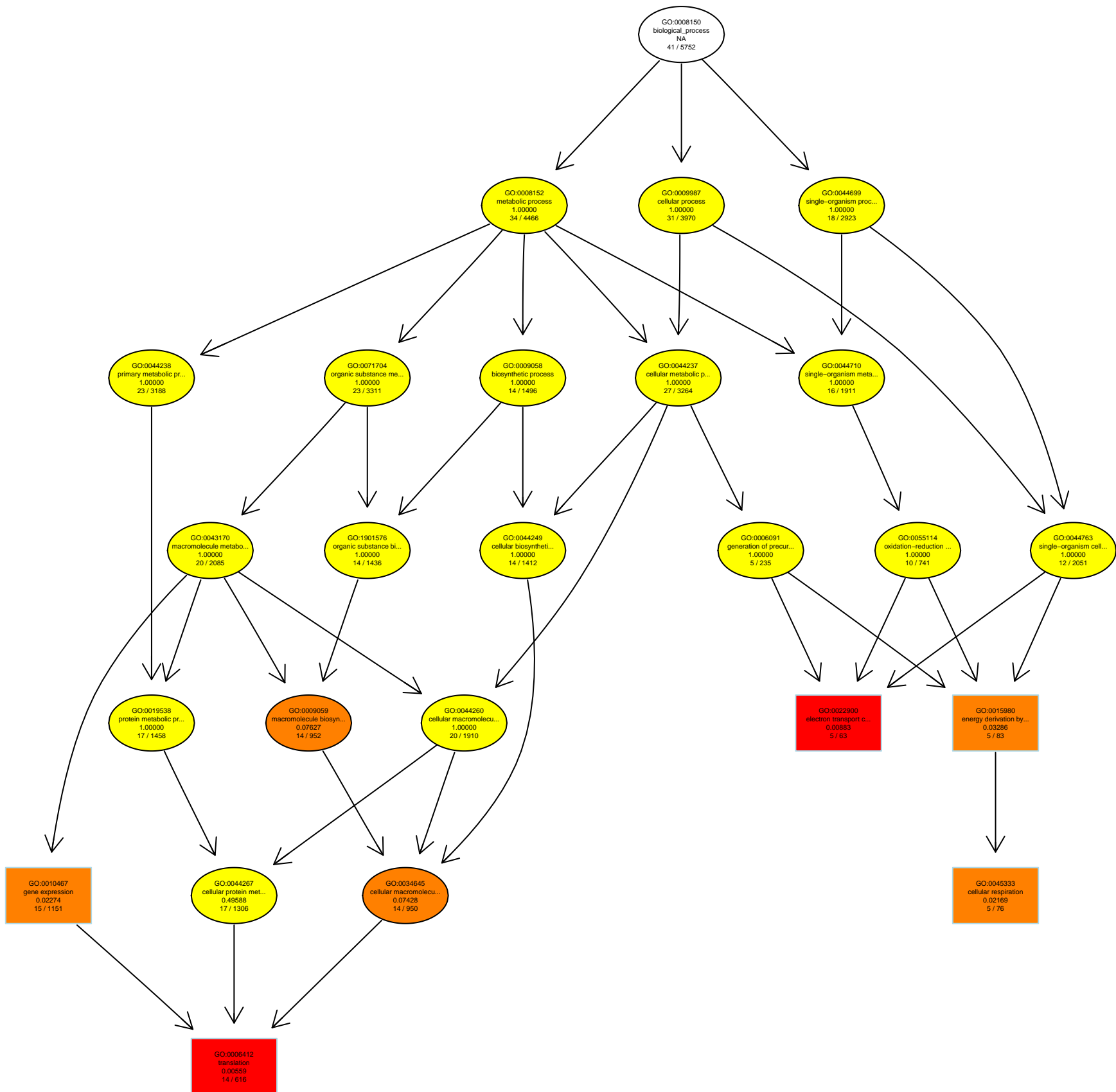

Supplement: S7 Fig — Coloring correlates with q-value. The lower the q-value, the more significant the enrichment. (PDF) [file pone.0193897.s007.pdf]

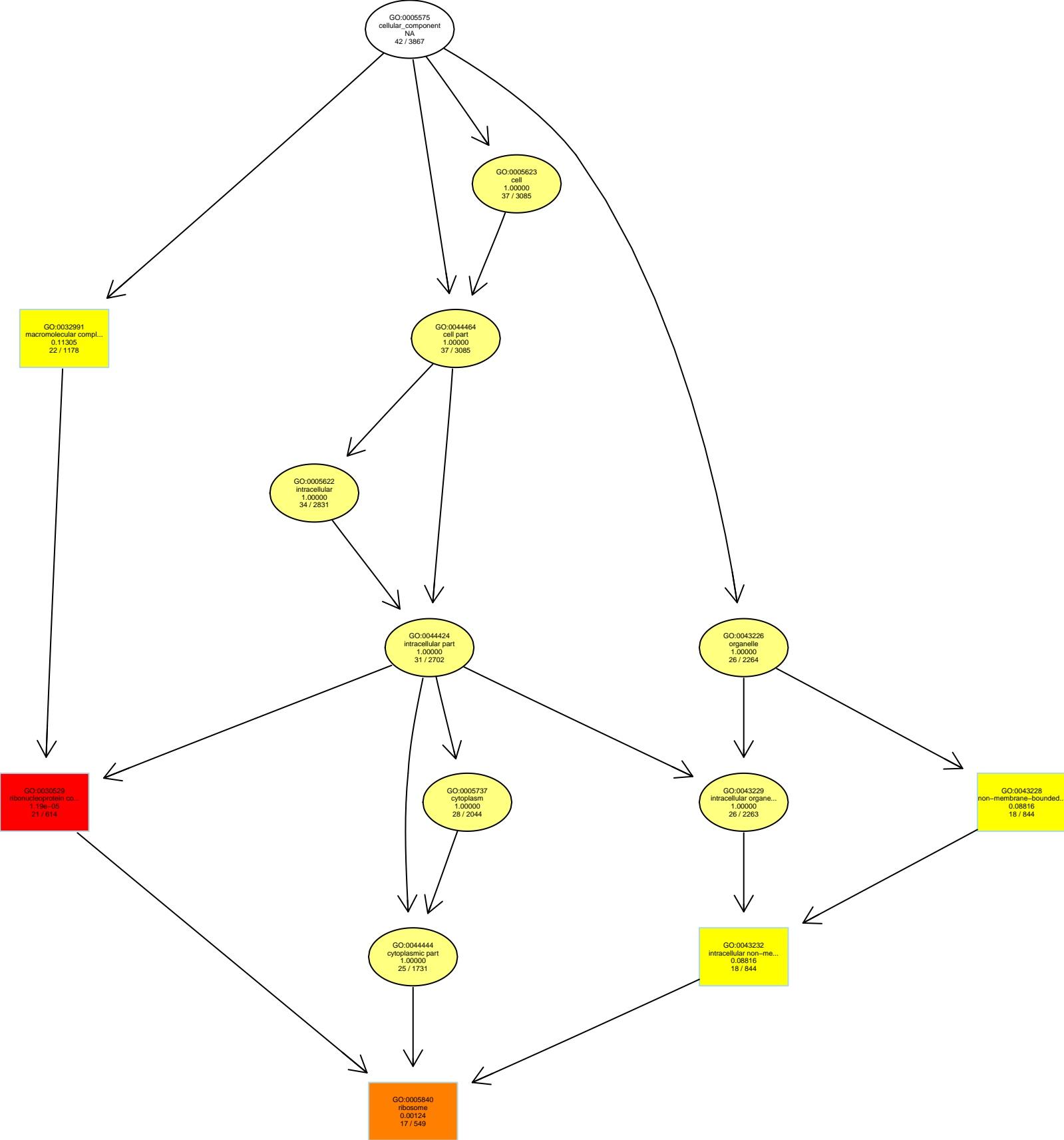

Supplement: S8 Fig — (PDF) [file pone.0193897.s008.pdf]

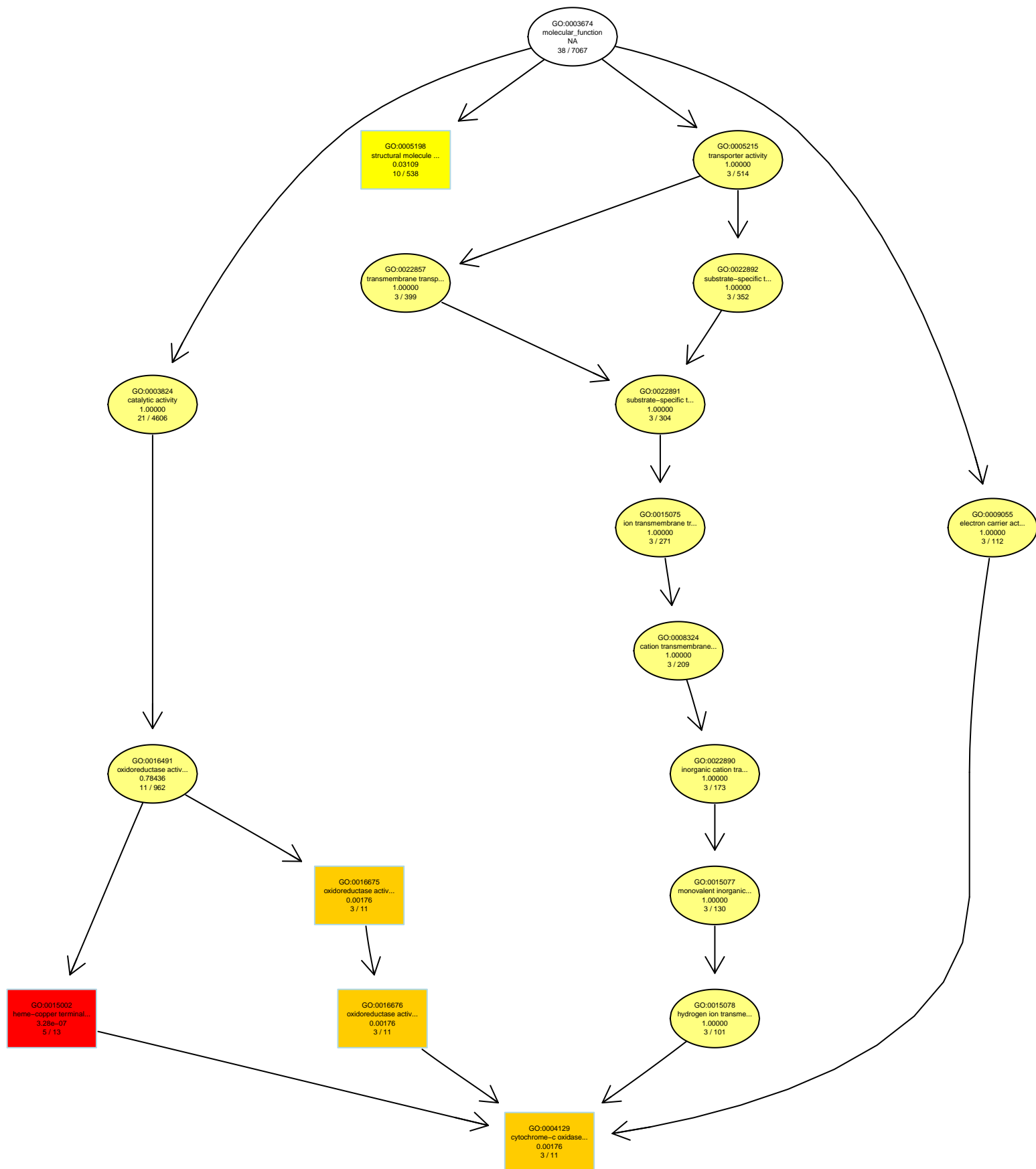

Supplement: S9 Fig — (PDF) [file pone.0193897.s009.pdf]

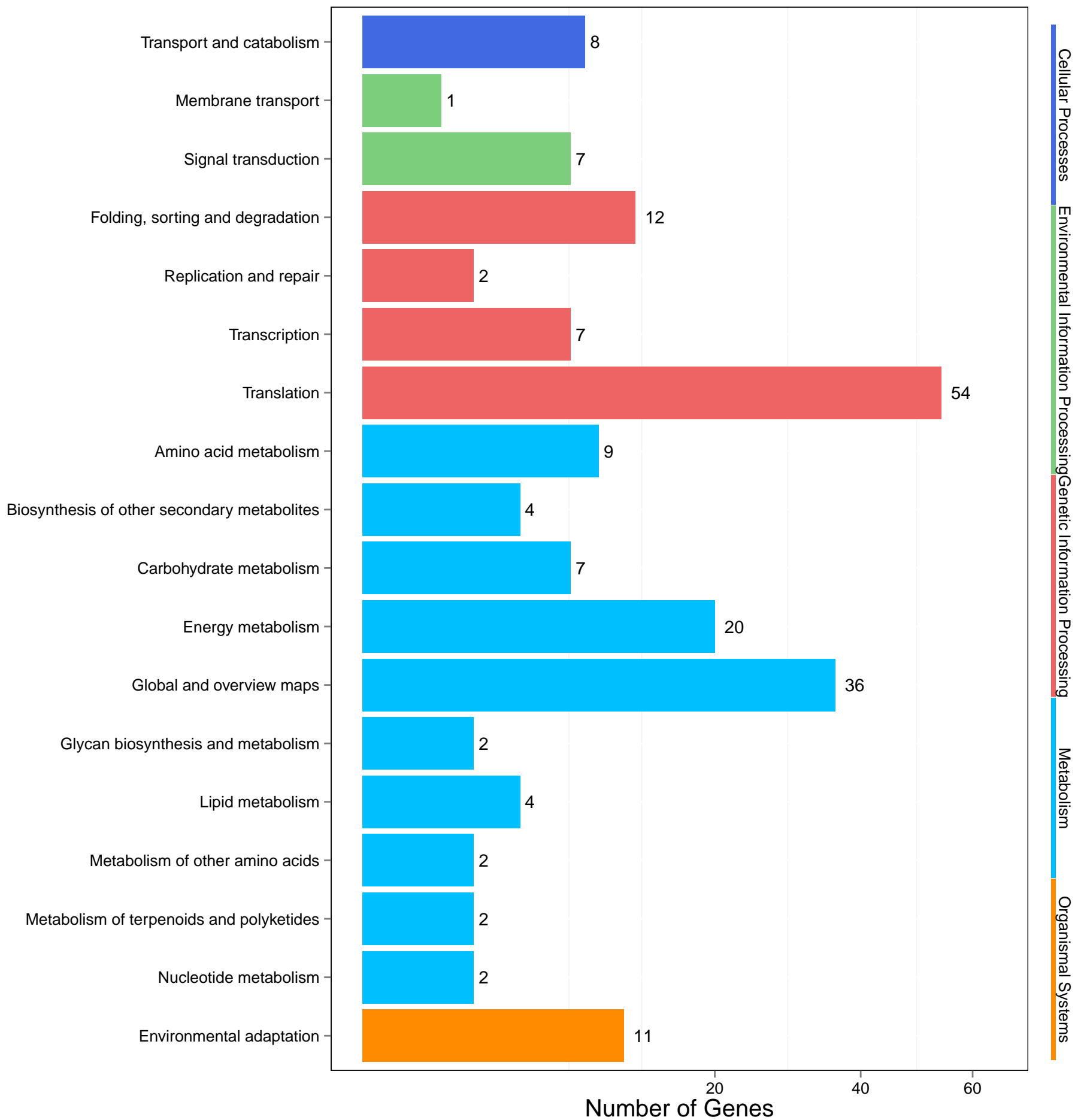

Supplement: S10 Fig — (PDF) [file pone.0193897.s010.pdf]

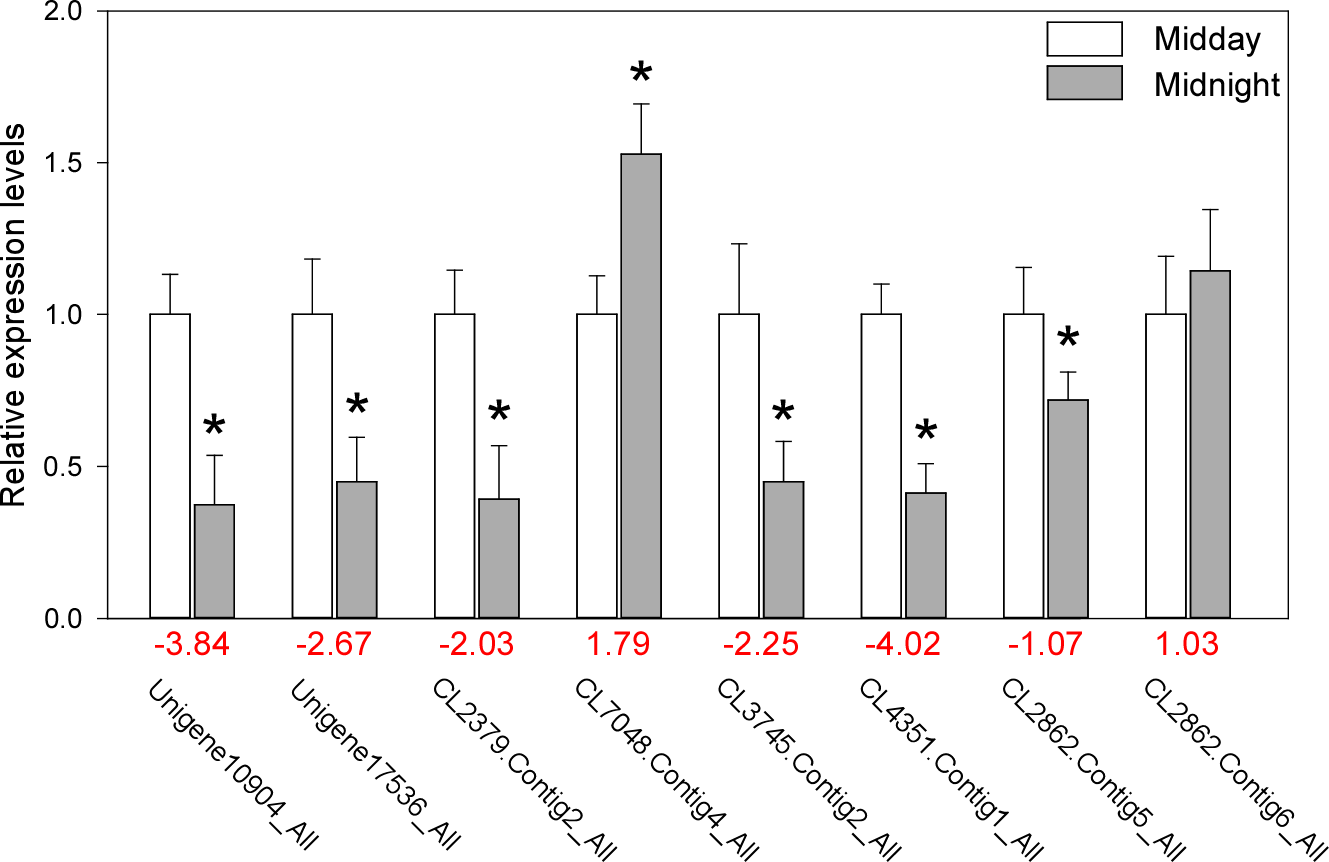

Supplement: S12 Fig — Asterisks indicate significant differences (P < 0.01; Student’s t-test). Red numbers under the bar graphs are log2-fold change values of each gene acquired in the transcriptome analysis. Three independent replicates were performed for midday and midnight (mean ± SD). (TIF) [file pone.0193897.s012.tif]

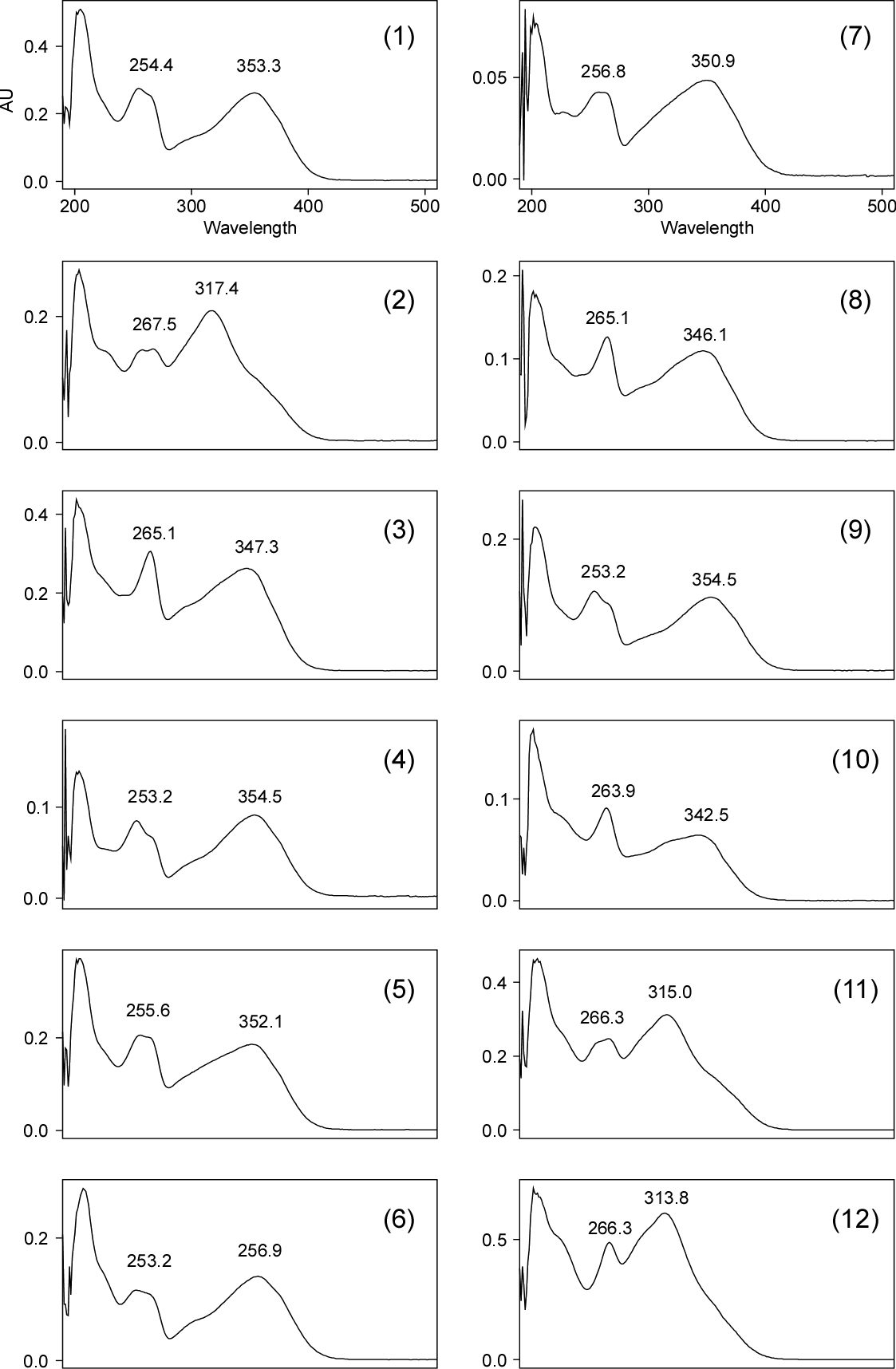

Supplement: S13 Fig — The wavelengths of two absorption peaks are marked. (TIF) [file pone.0193897.s013.tif]
