# Supplementary material for: Comprehensive transcriptome analysis and flavonoid profiling of Ginkgo leaves reveals flavonoid content alterations in day–night cycles
Source: PLoS One. 2018 Mar 1;13(3):e0193897. doi: 10.1371/journal.pone.0193897 (PMC5833276; doi:10.1371/journal.pone.0193897)
Supplement: S1 Table — (PDF) [file pone.0193897.s014.pdf]

Gene names	Primer sequences	Efficency	R2
GbCHS (CL1886.Contig5_All)	AACAAGTAAACCCATTCATACATCC<br>ATTTGAAATCGCAATACCAACC	95%	0.996
GbCHI (CL5281.Contig2_All)	CGGAAACTGCAAACGTTGTG<br>TGCTTGCTTCAACACTTGCA	97%	0.998
GbFLS (Unigene22251_All)	AGCCACGCACACTGTAATGG<br>GGCGGCTTTCTGCAACATAT	92%	0.991
GbF3H (CL2379.Contig4_All)	GGCCCAAAGTGGCGTACA<br>TCCGGCCAGTGAGATTATGG	95%	0.993
GbMYBF2 (Unigene12386_All)	TGAAACGGGGCTTACTGTCT<br>GTCGATTCCCATTTGCCGAA	93%	0.993
GbGAPDH	TAGGAATCCCGAGGAAATACC<br>TTCACGCCAACAACGAACATG	96%	0.996
Unigene10904_All	GCAAGGGCTTAGATCTACACT<br>ATTGCCCTTAAAATAGCTCGT	91%	0.995
Unigene17536_All	CAGTTATCTGCATTCACGAAC<br>CTGCTGCTGTTGATACATT	92%	0.998
CL2379.Contig2_All	TCGAACTGTTTGTTTATGTGCTT<br>CATGGAACATACTACAATCGC	91%	0.998
CL7048.Contig4_All	CAACAACATTAACTAAGCCAT<br>TTTTGAAGCATTCGTAGCCAT	93%	0.99
CL4351.Contig1_All	CGCTTTTCCCGTATCGAGA<br>CTTGCCCACTAATTCAACC	94%	0.997
CL2862.Contig5_All	GGCTCCATACCCCTAGTCCA<br>TCATCAAACTTGGTTGGCACA	93%	0.998
CL2862.Contig6_All	TATATTCGCCAGTGTCGTTG<br>GCCCAATGGATTTTAGCAA	92%	0.991
