# Supplementary material for: Comprehensive transcriptome analysis and flavonoid profiling of Ginkgo leaves reveals flavonoid content alterations in day–night cycles
Source: PLoS One. 2018 Mar 1;13(3):e0193897. doi: 10.1371/journal.pone.0193897 (PMC5833276; doi:10.1371/journal.pone.0193897)
Supplement: S2 Table — (PDF) [file pone.0193897.s015.pdf]

Sample	Total Number	Total Length	Mean Length	N50	N70	N90	GC(%)
D-1	66782	58482035	875	1695	999	308	42.18
D-2	73512	64064338	871	1718	990	302	42
D-3	78121	66650917	853	1726	953	293	41.88
N-1	72687	62334556	857	1686	953	300	42.22
N-2	77543	67050809	864	1751	968	298	41.89
N-3	71240	61962494	869	1695	972	307	42.29

N50: a weighted median statistic in which 50% of the total length is contained in transcripts greater than or equal to this value. GC (%): the percentage of G and C bases in all transcripts.
